# Supplementary material for: Content-rich biological network constructed by mining PubMed abstracts
Source: BMC Bioinformatics. 2004 Oct 8;5:147. doi: 10.1186/1471-2105-5-147 (PMC528731; doi:10.1186/1471-2105-5-147)
Supplement: Additional File 2 — The original results of the above study (non-essential files are deleted to keep the file size under the limit set by BMC bioinformatics). [file 1471-2105-5-147-S2.bz2 › chilibotAdditionalFile2/dip05/319ID10576729E2440/html/ATPA_ATPG.html]

 


 **ATPA** and **ATPG** 
  
Found 10 abstracts in PubMed, retrieved 05.  
 

 What does Google say? 
 PDF only 
| .edu only 

---

**Interactive relationship** (e.g. stimulation, inhibition, etc)

**Neutral relationship**- On tissue prints of cryostat sectioned buds, antibodies recognizing subunit A of the mitochondrial ATPase  **ATPA**  localized very differently from antibodies recognizing subunit II  [ **ATPG** ]  of the cytochrome oxidase COXII, which indicated that mitochondria in the same tissue could differentially express mitochondrially encoded proteins.  Ref: 12244222 Plant Cell< MedlineTA>Plant Cell,

**Non-interactive relationship** (e.g. studied together, co-existance, homology, etc.)

- While we were unable to create a deletion in the  **atpA**  gene, the plasmid pORI19 was inserted in a region between  **atpA**  and  **atpG**  to reduce, rather than eliminate, expression of the downstream genes.  Ref: 11016603 Int J Food Microbiol, 2000
- The C. thermoaceticum atp operon contained nine genes arranged in the order atpI i, atpB a, atpE c, atpF b, atpH delta,  **atpA**  alpha,  **atpG**  gamma, atpD beta, and atpC epsilon.  Ref: 9171425 J Bacteriol, 1997
- Eight genes atpI, atpB, atpE 1, atpE 2, atpE 3, atpF, atpH, and  **atpA**  upstream of and contiguous with the previously described genes  **atpG** , atpD, and atpC were cloned from chromosomal DNA of Acetobacterium woodii.  Ref: 10567365 J Biol Chem, 1999
